# Supplementary figures and images for: An improved multipath video data communication in a vehicular delay-tolerant network
Source: PLoS One. 2022 Sep 16;17(9):e0273751. doi: 10.1371/journal.pone.0273751 (PMC9480984; doi:10.1371/journal.pone.0273751)

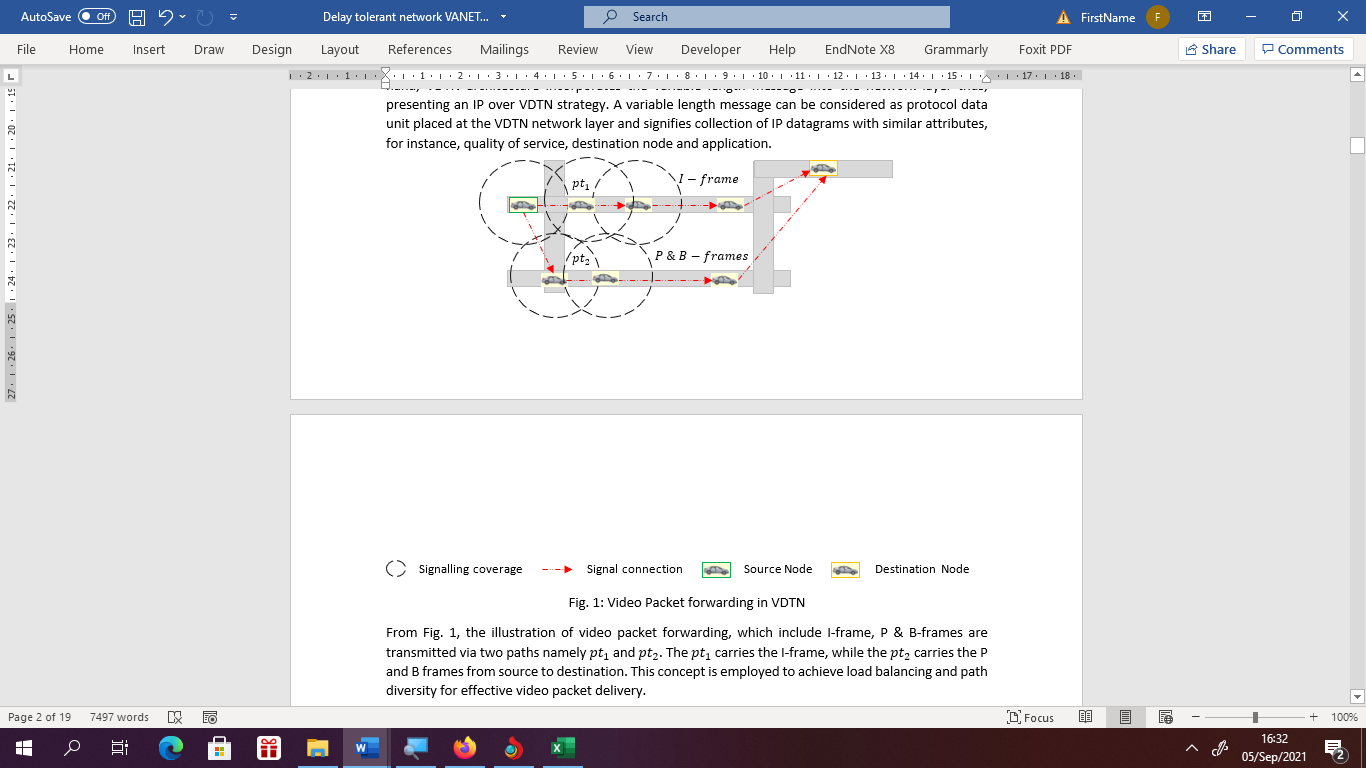


S1 Fig. 1: Video Packet forwarding in VDTN

Supplement: S1 Fig — (DOCX) [file pone.0273751.s001.docx]

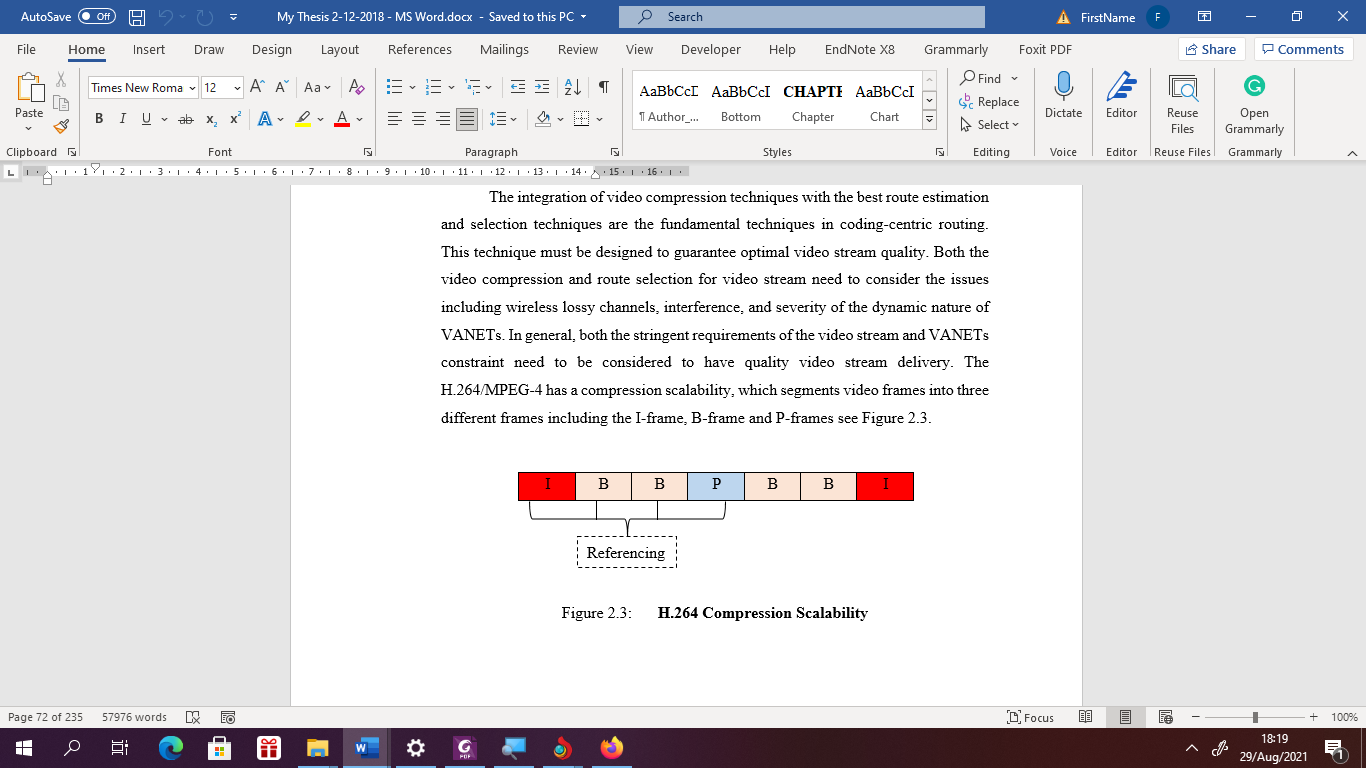


S2 Fig. 2: Video frames with compression scalability

Supplement: S2 Fig — (DOCX) [file pone.0273751.s002.docx]

`
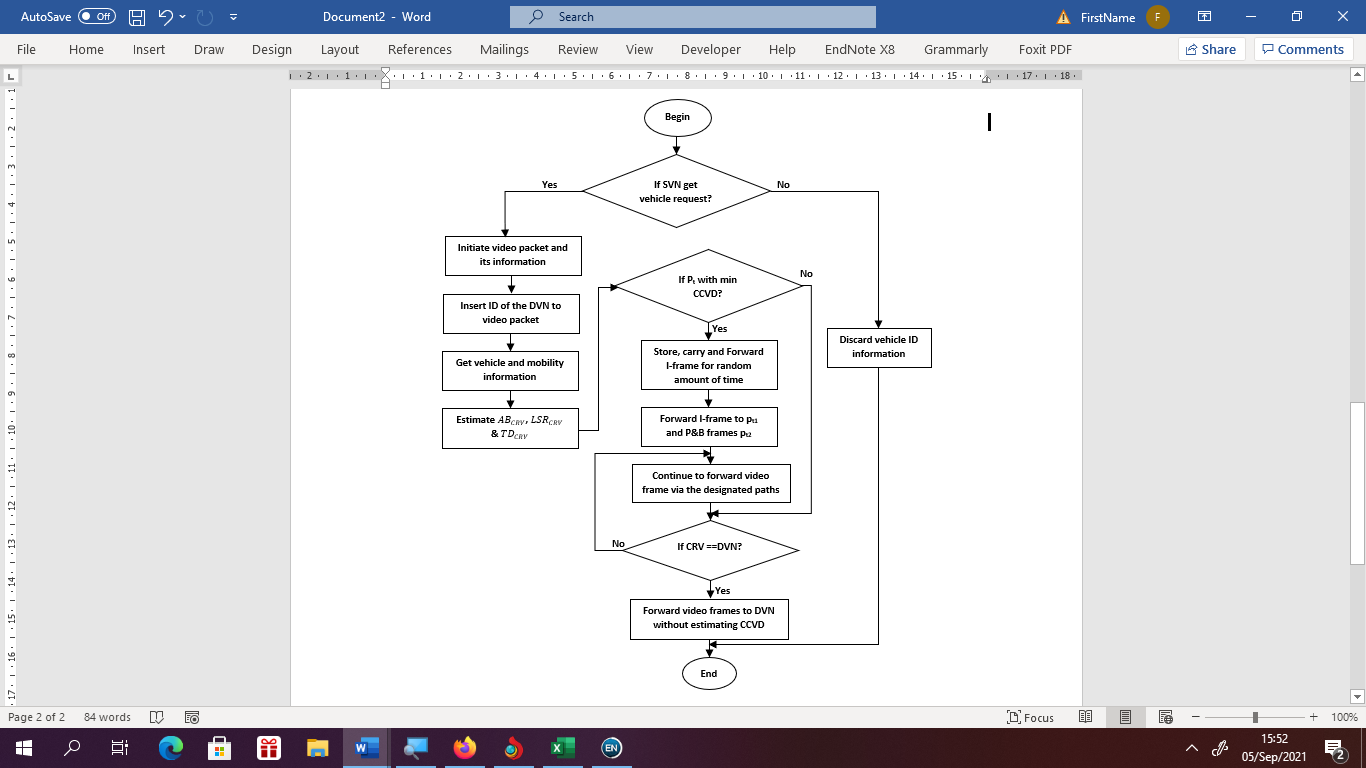


S4 Fig. 4: Video data forwarding flowchart

Supplement: S4 Fig — (DOCX) [file pone.0273751.s004.docx]

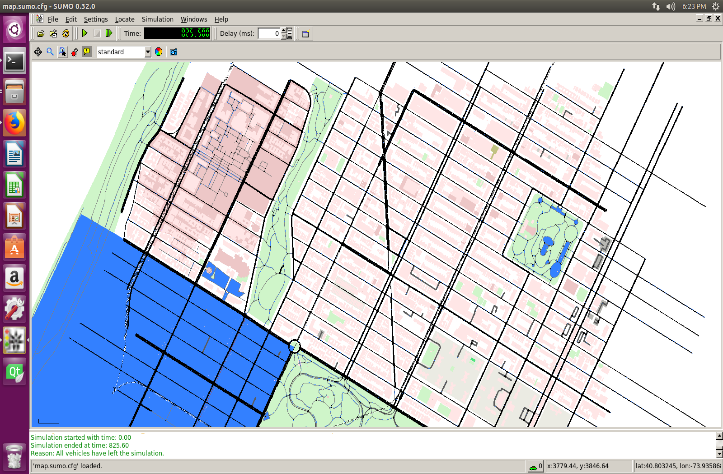


S5 Fig. 5: Simulation area based on Manhattan city map

Supplement: S5 Fig — (DOCX) [file pone.0273751.s005.docx]
