# Supplementary material for: An improved multipath video data communication in a vehicular delay-tolerant network
Source: PLoS One. 2022 Sep 16;17(9):e0273751. doi: 10.1371/journal.pone.0273751 (PMC9480984; doi:10.1371/journal.pone.0273751)
Supplement: S3 Fig — (DOCX) [file pone.0273751.s003.docx]

| Position(x, y) | Speed (v) | Road ID | No. Hop | Timestamp | HM count | Direction |
| --- | --- | --- | --- | --- | --- | --- |

S3 Fig. 3: Hello packet format
