# Supplementary material for: An improved multipath video data communication in a vehicular delay-tolerant network
Source: PLoS One. 2022 Sep 16;17(9):e0273751. doi: 10.1371/journal.pone.0273751 (PMC9480984; doi:10.1371/journal.pone.0273751)
Supplement: S6 Fig — (DOCX) [file pone.0273751.s006.docx]

S6 Fig. 6: PLR based on different video packet sending rates
