# Supplementary material for: An improved multipath video data communication in a vehicular delay-tolerant network
Source: PLoS One. 2022 Sep 16;17(9):e0273751. doi: 10.1371/journal.pone.0273751 (PMC9480984; doi:10.1371/journal.pone.0273751)
Supplement: S1 Algorithm — (DOCX) [file pone.0273751.s014.docx]

S1 Algorithm 1 Information gathering phase

**Algorithm 1** Information gathering phase

1: $\mathbf{Procedure}CreateHello(p_{i})$

2: $\mathbf{If}HelloTimeout elapsed \mathbf{Then}$

3: $\mathbf{Create} HelloMessage(HM)$

4: $\mathbf{Input} ID of p_{i}\mathrm{to}HM$ // insert the information of the hello message

5: $Input all info. of p_{i}\mathrm{to}HM$ // *p_i_*is the vehicular node

6: $\mathbf{Broadcast}HM with CurTime$

7: $\mathbf{Set}\mathrm{NewTimeout}$

8: $\mathbf{End if}$

9: $\mathbf{End Procedure}$

10: $\mathbf{New Procedure}$

11: $\mathbf{Procedure}\mathrm{ReceiveHello}\mathbf{(}p_{i}\mathbf{,}HM\mathbf{)}$

12: $\mathbf{If}HM\mathrm{received}\mathbf{Then}$

13: $\mathbf{If}ID\mathrm{of}p_{s} is not in ITN\mathrm{of}p_{r}\mathbf{Then}$// *p_s_* is the sending vehicle and pr is the receiving vehicle

14: $\mathbf{Create}NewRecord (Fresh)$ // generate a fresh record in the table

15: $\mathbf{Input} all info. of p_{s} to the ITN$ // insert senders’ information to the table

16: $\mathbf{Else}$

17: $\mathbf{Update}ID\mathrm{in}ITN$ //update the information in the table

18: $\mathbf{Update}all info. of p_{s}\mathrm{in}ITN$

19: $\mathbf{End if}$

20: $\mathbf{Else}$

21: $\mathbf{Discard (}HM\mathbf{)}$

22: $\mathbf{End if}$

23: $\mathbf{End Procedure}$
