# Supplementary material for: An improved multipath video data communication in a vehicular delay-tolerant network
Source: PLoS One. 2022 Sep 16;17(9):e0273751. doi: 10.1371/journal.pone.0273751 (PMC9480984; doi:10.1371/journal.pone.0273751)
Supplement: S2 Algorithm — (DOCX) [file pone.0273751.s015.docx]

S2 Algorithm 2 Video data forwarding based on communication cost

**Algorithm 2.** Video data forwarding based on communication cost

1. $\mathbf{Initiate:}video packet$
2. $\mathbf{Initiate:}\mathrm{videopktid}$
3. $\mathbf{Insert:}videopkttp= I\_frame, P\_frame, B\_frame$
4. $\mathbf{Insert:}ID\mathrm{of}DVN when forwarding videopkt$
5. $\mathbf{Output:} Forward videopkt from SVN\mathrm{to}DVN$
6. $\mathbf{Procedure}VideoDataForwarding(p_{i}, data)$
7. $\mathbf{If} video request received by SVN\mathbf{Then}$
8. $\mathbf{Obtain} speed, vehicle-id, direction, timestamp, position$
9. $\mathbf{Compute}Eq. (3)$
10. $\mathbf{Compute} Eq. (5)$
11. $\mathbf{Compute}Eq. (8)$
12. $\mathbf{Obtain}CCVD value of each C-NFV$
13. $\mathbf{Identify} suitable first two \left( p_{i\left( s \right)} RVN \right)$
14. $\mathbf{Input}ID for the selected first two (RVN=P_{t1},P_{t2})$
15. $\mathbf{Else}$
16. $\mathbf{Discard}p_{i(s)} ID$
17. $\mathbf{If}p_{i}Max({AB}_{CRV}\&\&{LSR}_{CRV}\&\&{TD}_{F}) \mathbf{Then}$
18. $\mathbf{Store}\mathrm{videopkttp}\left( I_{frame} \right)$
19. $\mathbf{Forward}\mathrm{videopkttp}\left( I_{frame} \right)\& videopktid to P_{t1}\& otherp_{i}in same path$
20. $\mathbf{Forward}\mathrm{videopkttp}\left( P_{frame},B_{frame} \right)\mathrm{to}P_{t2}\& otherp_{i}$
21. $\mathbf{Waitand Carry} for RandAmtTime$
22. $\mathbf{Retransmit}\mathrm{videopkttp}\left( I\_frame \right)\mathrm{via}P_{t1}$
23. $\mathbf{Endif}$
24. $\mathbf{Else}$
25. $\mathbf{If}P_{t1},P_{t2} already receives videopkttp \mathbf{Then}$
26. $\mathbf{Compute}\mathrm{the}CCVD$
27. $\mathbf{Select}qualified relay node based on CCVD$
28. $\mathbf{Forward} videopkttp from P_{t1}, P_{t2} to their NFVs$
29. $\mathbf{Input}ID of qualified NFVs to TNI\mathrm{of}P_{t1} , P_{t2}$
30. $\mathbf{End if}$
31. $\mathbf{Else}$
32. $\mathbf{If}p_{i}not optimal \mathbf{Then}$
33. $\mathbf{Discard} (videopkt p_{i(s)})$
34. $\mathbf{End if}$
35. $\mathbf{Else}$
36. $\mathbf{Forward}\left( \mathrm{videopkt} \right) to qualified NFV$
37. $\mathbf{If} NFV==DVN\mathbf{Then}$
38. $\mathbf{Forward}\left( \mathrm{videopkt} \right)\mathrm{to}DVN without computing CCVD$
39. $\mathbf{Check} for duplicate I-Frame with the same ID and Discard one$
40. $\mathbf{Else}$
41. $\mathbf{Forward}\left( \mathrm{videopkt} \right)\mathrm{to}NFV$
42. $\mathbf{End if}$
43. $\mathbf{End if}$
44. $\mathbf{End Procedure}$
