# Supplementary material for: An improved multipath video data communication in a vehicular delay-tolerant network
Source: PLoS One. 2022 Sep 16;17(9):e0273751. doi: 10.1371/journal.pone.0273751 (PMC9480984; doi:10.1371/journal.pone.0273751)
Supplement: Attachment — Submitted filename: Response to Reviewers.docx [file pone.0273751.s016.docx]

**Manuscript ID:** PONE-D-21-30574

**Paper Title:** Improved Multipath video data communication in Vehicular Delay Tolerant Network.

**To:** PLOS ONE Editor

**Re:** Response to Reviewers

Dear Editor,

Thank you for allowing a resubmission of our manuscript, with an opportunity to address the reviewers’ comments.

We are uploading:

(1) Our point-by-point response to the comments (below) (response to reviewers).

(2) An updated manuscript with Red Font Colour indicating changes, and

(3) A clean updated manuscript without highlights.

Best regards,

Dr. Khaled H Almotairi

Assistant Professor

Email: [khmotairi@uqu.edu.sa](mailto:khmotairi@uqu.edu.sa)

**REVIEWER 1- COMMENTS**

**Comment 1**

The contribution and motivation of this paper are not clear. What is the motivation to design the store-carry-forward with the consideration of multipath video data transmission? What are the advantages of estimating the video data communication cost for relay nodes? The authors are invited to give a detailed discussion on these issues.

**Response to comment 1**

First, we would like to express our sincere gratitude to the reviewer for the valuable comments. In order to strengthen the motivation of store-carry-forward with the consideration of multipath video data transmission and the advantage of estimating the video data communication cost for relay node, the following enhancements have been made to the revised manuscript. With the enhancement, we hope the improvement will be considered.

The contribution and motivation of this paper have been discussed in the Introduction section (Section 1). In addition, the advantage of estimating video data communication cost has also been highlighted in Section 1 of the revised manuscript.

**Comment 2**

The performance comparison is presented based on the simulation results. It is insufficient to show the superiority of the proposed MVDTN scheme by simulation. Some experiment results should be given to show the implementability of the designed MVDTN.

**Response to comment 2**

Thank you for the comment, creating a real-life outdoor testbed for experimentation of vehicular network is expensive and difficult due to the cost of deployment. Thus, in this study, we used simulation approach to implement our proposed MVDTN. Meanwhile, experiment results have been provided in the performance evaluation section (Subsection 4.1).

**Comment 3**

This manuscript looks like a technical manual rather than a scientific article. The authors should give an intensive theoretical analysis on the proposed results.

**Response to comment 3**

To honour your comment, the whole of the results analysis subsection has been revised to comply with the intensive theoretical analysis of the obtained results.

**Comment 4**

The readability should be improved of this paper should be improved. There are some typos throughout the paper that should be corrected.

**Response to comment 4**

Thank you very much for the valuable comment. All the typographical and grammatical errors have been corrected throughout the manuscript.

**REVIEWER 2- COMMENTS**

**Comment 1**

This paper studies how to transmit video efficiently without losing frames in vehicular network. The problem selection has practical significance. The language of the article is fluent, and the experiment is sufficient. But the innovation is not strong. Can you discuss what is the essential difference between and distributed storage?

**Response to comment 1**

First, we would like to express our sincere gratitude to the reviewer for the valuable comment. To honour your suggestion, the term distribution storage is based on infrastructure that can partition data across multiple physical servers, which is usually across more than one data centres.

On the other hand, the store-carry-forward strategy is where each node stores data packets in the buffer, and whenever the node meets another node, then it forwards the duplicated data packet.

**REVIEWER 3- COMMENTS**

**Comment 1**

First of all, the title of this paper is “An Improved Multipath video data communication in Vehicular Delay Tolerant Network”. From the author's abstract section, it is difficult to see where the improvements proposed by the author are. In fact, the author doesn’t give the current research hot issues in the first few sentences of the abstract.

**Response to comment 1**

First, we would like to express our sincere gratitude to the reviewer for the valuable comment, the improvement in this paper is based on the addition of store-carry-forward strategy in multipath data forwarding over vehicular network. In addition, the recent research hot issues are highlighted in the abstract section.

**Comment 2**

The author's contribution is too few and the description is unclear. It is difficult for me to understand the author's second and third contribution points as two complete ones.

**Response to comment 2**

Thank you for the observation, the second contribution of this paper is focused on calculating communication cost, which are based on three parameters namely, link stability rate, bandwidth availability and transmission delay. On the other hand, the third objective is based on transmitting video frames, which are categorized into I-frame P-frame and B-frame. Priority is given to the I-frame, which is the important frame and is forwarded through the path with the best cost. While the P and B-frames are forwarded via another path.

**Comment 3**

In the related work section, the author's description is too redundant. In fact, the related work is a summary of previous work. It is commendable that the author selected several points in this part and cited a large number of literatures for analysis. However, some of the author's concluding remarks are too redundant. For example, on page 6, the author explains some problems in literature [29] in more than 10 lines which is too detailed and unnecessary.

**Response to comment 3**

To reduce the redundant description, the related work section has been revised and corrections have been made (See Page 6 of the manuscript).

**Comment 4**

The author made a lot of grammatical and formatting errors in his article. For example, there is nothing after the 'and' of the author's contribution; As on page 6, lines 151 and 154, the author quotes [?]. On Line 217 of page 9, the meaning of “Variable f represents” is unknown. The author had better check the whole paper carefully to correct grammatical errors, which appeared too many times and even affected reading.

**Response to comment 4**

Thank you very much for the informative comment. All the typographical and grammatical errors have been corrected throughout the manuscript.
