# Supplementary material for: An improved multipath video data communication in a vehicular delay-tolerant network
Source: PLoS One. 2022 Sep 16;17(9):e0273751. doi: 10.1371/journal.pone.0273751 (PMC9480984; doi:10.1371/journal.pone.0273751)
Supplement: Attachment — Submitted filename: Response to Reviewers.docx [file pone.0273751.s017.docx]

**Manuscript ID:** PONE-D-21-30574

**Paper Title:** An improved multipath video data communication in a vehicular delay Tolerant Network.

**To:** PLOS ONE Editor

**Re:** Response to Reviewers

Dear Editor,

Thank you for allowing a resubmission of our manuscript, with an opportunity to address the reviewers’ comments.

We are uploading:

(1) Our point-by-point response to the comments (below) (response to reviewers).

(2) An updated manuscript with Red Font Colour indicating changes, and

(3) A clean updated manuscript without highlights.

(4) An image file (File of figures).

Best regards,

Dr. Khaled H Almotairi

Assistant Professor

Email: [khmotairi@uqu.edu.sa](mailto:khmotairi@uqu.edu.sa)

**REVIEWER 1- COMMENTS**

**Comment 1**

Reviewer #1: The paper has been improved. All the previous suggestions have been well considered and thoroughly implemented in this revised paper. Hence, this paper could be accepted for publication now.

**Response to comment 1**

Thank you very much for recommending acceptance of the paper. In addition, we would like to mention our sincere appreciation for your efforts in improving the manuscript.

**REVIEWER 3- COMMENTS**

**Comment 1**

Reviewer #3: After the revision of the original version, the following problems still exist in this paper:
1. This paper involves many algorithms, but the part of the derivation is too concise. I think it is necessary to explain the algorithms properly, which can help readers better understand them. It is suggested to give detailed and clear explanation.

**Response to comment 1**

Thank you for the valuable comments. To handle your suggestion, a detailed and comprehensive explanation of the algorithms have been provided (see Pages 11, 13 and 14). In addition, some of the steps required in the derivations have been added (see Page 9)

**Comment 2**

2.There are some wrong fonts in the paper. For example, on page 10, line 257, "HM" should be italicized. Please check the whole paper and correct similar mistakes carefully.

**Response to comment 2**

Thank you for the observation, “HM” and other variable terms have been corrected throughout the manuscript (see Pages 10 and 11).

**Comment 3**

3.The format of the references is not uniform in the paper. For example, in references 19 and 32, "IEEE" is lowercase while it is uppercase in other references. The authors should modify the format of references carefully.

**Response to comment 3**

We appreciate your comments, the whole of the reference section has been corrected in order to be uniform in terms of format. For example, see reference 19 and 32, thank you.

**REVIEWER 4- COMMENTS**

**Comment 1**

Reviewer #4: The authors have revised the manuscript. The overall structure of the paper is clear. The simulation results show that the proposed scheme is superior to the baseline approaches. To make this paper accepted, I think relevant content still need to be improved, which is shown below.
1. This paper involves the derivation of many formulas, but some derivations are too brief, such as page 9. It is suggested to add intermediate steps appropriately.

**Response to comment 1**

Thank you very much for the important comments. To acknowledge this comment, the required intermediate steps for derivation of the formulas have been included. For example, see Pages 8 and 9 of the manuscript.

**Comment 2**

2. In the last part of the figure, it's best to remove the outer border of the picture to make the picture cleaner.

**Response to comment 2**

We appreciate the precise comment, the outer border of the figure has been removed to make the figure clearer (see all figures in the manuscript).

**Comment 3**

3. The format of the references is not uniform in the paper. The authors should modify the format of references carefully.

**Response to comment 3**

Thank you, the whole of the reference has been formatted and corrected in order to be uniform in the whole of the manuscript (see Pages 25-27).

**Comment 4**

4. There are some punctuation mistakes. For example, when introducing contribution points on page 3, the first point ends with a comma and the other two points end with a full stop, hoping that the author can correct similar mistakes.

**Response to comment 4**

We appreciate your observation; all punctuation mistakes have corrected throughout the manuscript. For example, see Page 3 of the manuscript.
